# Severe autoimmune diffuse alveolar hemorrhage in children; early diagnosis and initiation of therapeutic plasma exchange may improve clinical outcomes

**DOI:** 10.3389/fped.2026.1799535

**Published:** 2026-04-07

**Authors:** Jack Steven Thomas, Dai Kimura

**Affiliations:** 1 Department of Pediatrics, University of Tennessee Health Science Center/Le Bonheur Children's Hospital, Memphis, TN, United States; 2 Department of Pediatrics, Division of Critical Care Medicine, Apheresis Service, University of Tennessee Health Science Center/Le Bonheur Children’s Hospital, Memphis, TN, United States

**Keywords:** ANCA-associated vasculitis, diffuse alveolar hemorrhage, extracorporeal membrane oxygenation, systemic lupus erythematosus, therapeutic plasma exchange

## Abstract

**Introduction:**

Diffuse alveolar hemorrhage (DAH) is a rare but life-threatening pulmonary complication in children, presenting with hemoptysis, anemia, diffuse infiltrates, and respiratory failure. Autoimmune diseases such as systemic lupus erythematosus (SLE) and ANCA-associated vasculitis (AAV) account for 30%–40% of pediatric DAH cases. Pediatric evidence is limited to case reports and small series. We aimed to characterize the clinical course, management, and outcomes of pediatric patients with autoimmune DAH requiring intensive care.

**Methods:**

We conducted a retrospective cohort study at a single tertiary pediatric institution, identifying 6 patients admitted to the ICU from 2013 to 2024 with DAH secondary to SLE or AAV.

**Results:**

All six patients (5 females; age 14–17) were critically ill. Five required MV (median 18 days, IQR: 18–25), two required high-frequency oscillatory ventilation (HFOV) (6, 10 days), and three required VV-ECMO (8, 9, and 45 days). Four had new-onset autoimmune diagnoses on admission. All received high-dose corticosteroids and therapeutic plasma exchange (TPE) (mean 6.5 ± 2.6 sessions); adjunctive therapies included cyclophosphamide, rituximab, IVIG, mycophenolate mofetil, and hydroxychloroquine. TPE was initiated within 24 h of DAH diagnosis in most cases. Two patients with less severe presentations had delayed initiation, and one progressed to HFOV. All patients on ECMO were successfully decannulated; one died from *Pseudomonal* sepsis post-decannulation. The remaining patients survived to discharge.

**Conclusion:**

Autoimmune DAH in pediatric patients carries high morbidity, often requiring advanced respiratory support and multimodal immunosuppression. Early diagnostics work up and initiation of TPE may improve outcomes. Further prospective pediatric study would be necessary to guide standardized treatment protocols.

## Introduction

Diffuse alveolar hemorrhage (DAH) is a rare but life-threatening condition characterized by bleeding into the alveolar spaces, presenting with acute hemoptysis, anemia, nonspecific respiratory symptoms, diffuse alveolar infiltrates on imaging, and hypoxemic respiratory failure—all of which may necessitate urgent intervention (1–3). Severe DAH is typically defined by oxygen saturation of ≤85% on room air or the need for mechanical ventilation. Autoimmune diseases such as anti-glomerular basement membrane (anti-GBM) disease, antineutrophil cytoplasmic antibody (ANCA)-associated vasculitis (AAV), antiphospholipid syndrome (APS), and systemic lupus erythematosus (SLE) are estimated to account for 30%–40% of DAH cases (4).

In severe cases, pediatric patients often require endotracheal intubation to protect the airway and mechanical ventilation with high positive end-expiratory pressure to provide a tamponade effect to the bleeding site (5) and, in some instances, extracorporeal membrane oxygenation (ECMO) for respiratory support (6). Treatment regimens for DAH associated with autoimmune disease in children are largely based on adult studies, or pediatric case reports and small cohort studies (7). The cornerstone of therapy includes high-dose or pulse-dose glucocorticoids, often in combination with additional immunosuppressive agents such as cyclophosphamide or rituximab (8–10).

Therapeutic plasma exchange (TPE)—also referred to as plasmapheresis or plasma exchange therapy—has been reported in several case series as an adjunctive treatment for autoimmune DAH in pediatric populations (7, 11–13). TPE is theoretically believed to remove pathogenic autoantibodies, immune complexes, and inflammatory mediators contributing to the disease process, and provide clotting factors to correct coagulopathy without fluid overload. However, recommendations regarding TPE use have evolved; recent American Society for Apheresis (ASFA) guidelines have become more conservative in their endorsement of TPE for DAH secondary to SLE or AAV (14). Although TPE carries inherent risks, DAH remains a severe and potentially fatal complication of autoimmune disease (15, 16), prompting ongoing debate about whether the potential benefits of TPE outweigh its risks—especially in critically ill patients requiring mechanical ventilation with high ventilator settings, high frequency oscillatory ventilation, or ECMO.

Mortality rate for adults with autoimmune DAH is historically high, with reported death rate from 24% to 80% (17–20), dependent on severity and underlying cause, but pediatric data are limited. In this study, we present a case series of six pediatric patients with DAH secondary to SLE or AAV treated with TPE at our institution between 2013 and 2024. These patients either initially presented with florid pulmonary symptoms consistent with DAH or progressed to severe disease requiring invasive interventions. We hypothesize that earlier initiation of TPE may improve morbidity and mortality outcomes in this high-risk pediatric population.

## Method

We performed a single institution retrospective cohort study analyzing the medical records of patients who were admitted to the intensive care unit (ICU) in our tertiary children's hospital. This study was approved by the University of Tennessee Health Science Center/Le Bonheur Children's Hospital Institutional Review Board prior to data collection. Patient eligibility required ICU admission between January 2013 and December 2024, and a diagnosis of DAH (ICD-10 R04.89) due to SLE (ICD-10 M32) or ANCA vasculitis/AAV (ICD-10 I77.82). The diagnosis of DAH was made with the following criteria: hemoptysis and/or new pulmonary infiltrates on chest radiography and/or anemia (DAH triad); bronchoalveolar lavage fluid with either being macroscopically hemorrhagic or presence of hemosiderin-laden macrophages on cytological analysis.

## Results

Six patients were admitted to our ICU with either nonspecific systemic or respiratory symptoms that progressed to DAH, or with DAH as their presenting symptom. All patients were ultimately diagnosed with an underlying autoimmune condition consistent with either SLE or AAV. The patients' mean age was 15.5 years (SD: 15.5 ± 1.4), with a female predominance (*n* = 5) (Table 1).

**Table 1:** Patient characteristics, presenting features, autoimmune diagnostic findings, and pre-admission immunomodulatory therapy.

Variable	Summary
Demographics	
Age, years	Mean ± SD: 15.5 ± 1.4
Female sex, *n* (%)	5 (83%)
Autoimmune diagnosis, *n* (%)	
SLE	3 (50%)
AAV	2 (33%)
Combined SLE and AAV	1 (17%)
New autoimmune diagnosis during admission, *n* (%)	4 (67%)
Notable labs on admission	
Anemia, *n* (%)	6 (100%)
Leukopenia, *n* (%)	3 (50%)
Thrombocytopenia, *n* (%)	1 (17%)
Hematuria ± proteinuria, *n* (%)	5 (83%)
Preceding symptoms	
Chest/back pain, *n* (%)	4 (67%)
SOB/cough, *n* (%)	6 (100%)
Rash, *n* (%)	3 (50%)
Polyarthralgia, *n* (%)	5 (83%)
Hemoptysis, *n* (%)	6 (100%)
Renal involvement, *n* (%)	5 (83%)
Renal biopsy performed, *n* (%)	2 (33%)*
Immunomodulators/medications prior to admission	
Prednisolone, *n* (%)	4 (67%)
CYC, *n* (%)	0 (0%)
MMF, *n* (%)	1 (17%)
HCQ, *n* (%)	1 (17%)

* Renal biopsy was deferred in three patients due to clinical instability despite suspected renal involvement.

Laboratory abnormalities, biopsy results, and preceding symptoms reflect findings at presentation or during hospitalization. Renal biopsy was deferred in Patients #1, #4, and #5 because of clinical instability, although renal involvement was suspected based on laboratory and clinical findings. SLE, systemic lupus erythematosus; ANCA, antineutrophil cytoplasmic antibodies; MPO, myeloperoxidase; PR3, proteinase 3; ANA, antinuclear antibody; DNA, deoxyribonucleic acid; GPA, granulomatosis with polyangiitis; AKI, acute kidney injury; CYC, cyclophosphamide; MMF, mycophenolate mofetil; SOB, short of breath; RBC, red blood cell.

### Clinical presentation and diagnosis

Four of the six patients had preceding symptoms suggestive of autoimmune disease in the months leading up to admission, but only two of these patients had been previously evaluated by pediatric rheumatology and initiated on immunosuppressive therapy, while the other two presented with primarily respiratory complaints (Table 1). The remaining four were followed by primary care providers and received treatment for vague symptoms (cough, myalgias, rash, etc.) before admission and received their formal autoimmune diagnosis during the index hospitalization. Pediatric rheumatology was consulted for all patients and involved in the evaluation and treatment plans during these hospitalizations.

The final diagnoses of their autoimmune condition were confirmed by a combination of serologic testing, biopsy results, and clinical correlation. Pertinent laboratory, diagnostic findings, and preceding symptoms are detailed in Table 1. Time from initial autoimmune diagnostic confirmation to hospital admission varied, with four patients confirming their autoimmune diagnosis during the current hospitalization. When available from chart review, the interval between hospital admission and confirmation of DAH ranged from the day of admission to several days later, reflecting the diagnostic challenges posed by nonspecific imaging findings and the need for bronchoscopy or catheter-based evaluation. Once DAH was confirmed, TPE was initiated within 24 h in four patients. All patients presented with hemoptysis, though the timing of DAH confirmation varied between patients. Five of the patients were found to have renal involvement.

### Respiratory support and clinical outcomes

All but one patient required endotracheal intubation and mechanical ventilation for acute respiratory failure and airway protection; one patient's maximum respiratory support was BiPAP (Table 2). Intubation occurred at or near the time of ICU admission in nearly all cases due to the severity of hemoptysis and concern for rapid decompensation. Five patients met criteria for ARDS, and two required escalations to high-frequency oscillatory ventilation (HFOV) for refractory hypoxemia and ongoing pulmonary bleeding. Three patients received inhaled tranexamic acid and Factor VII as adjunctive therapy for persistent hemoptysis. Two patients experienced complications of pneumomediastinum, with one patient developing it after TPE and ECMO initiation, and the other patient developing it after requiring higher ventilator settings, resulting in ECMO initiation. Three patients progressed to severe hypoxemic respiratory failure requiring venovenous ECMO. Five patients underwent bronchoscopy for evaluation of airway bleeding, and two patients required catheter-based evaluation for possible bronchial artery embolization (one patient; this intervention was unsuccessful, and for the other patient, they underwent bronchial artery embolization + bilateral internal mammary artery embolization). Before TPE initiation, all patients required packed red blood cell transfusions due to anemia from hemorrhage (mean 4.3 ± 2.5 units; Table 2). Three patients received platelet transfusions after initiation of TPE, administered for ongoing pulmonary bleeding with thrombocytopenia (two patients) or gastrointestinal bleeding (two patients).

**Table 2:** Respiratory support requirements, major clinical interventions, and outcomes.

Variable	Summary
ICU length of stay (days)	Median (IQR): 20.5 (20–34.5)
Hospital length of stay (days)	Median (IQR): 32.5 (29–46)
Direct ICU admission, *n* (%)	4 (67%)
Invasive mechanical ventilation, *n* (%)	5 (83%)
Duration of MV (days)	Median (IQR): 18 (18–25)
HFOV, *n* (%)	2 (33%)
HFOV duration (days)	6 and 10
VV-ECMO, *n* (%)	3 (50%)
Timing to cannulation, hospital day	Median (range): 4 (1–4)
ECMO duration, days	Median (range): 9 (8–45)
CVVH, *n* (%)	3 (50%); Median (range) days: 2 (1–77)
CVVH indication	Renal failure, uremia, and volume overload
Bronchoscopy, *n* (%)	5 (83%)
Catheterization, *n* (%)	2 (34%)
Adjunctive inhaled TXA, *n* (%)	3 (50%); Median (range), days: 17 (21–23)
Adjunctive inhaled Factor VII, *n* (%)	3 (50%); Median (range), days: 4 (3–6)
Packed RBC transfusion before TPE, *n* (%)	6 (100%); Mean ± SD, units: 4.3 ± 2.5
Platelet transfusion after TPE initiation, *n* (%)	3 (50%)
Survival to discharge, *n* (%)	5 (83%)

Details include the need for mechanical ventilation (MV), high-frequency oscillatory ventilation (HFOV), extracorporeal membrane oxygenation (ECMO), adjunctive hemostatic therapies, transfusion requirements, and ICU/hospital length of stay. HDGC, high-dose glucocorticoids; TXA, tranexamic acid; CVVH, continuous veno-venous hemofiltration; AWP, airway protection; HD, hospital day.

The duration of mechanical ventilation was 18 (median, IQR: 18–25) days, with HFOV for 6 and 10 days and ECMO for 8, 9, and 45 days. ICU length of stay was 20.5 (median, IQR: 20–34.5) days. One patient was briefly transferred to the acute-care floor before experiencing rapid clinical deterioration requiring ICU readmission and reintubation for acute hypoxemic respiratory failure within 12 h. One patient developed persistent vasoplegic shock with *Pseudomonas* bacteremia, bleeding from the gastrointestinal tract, and multiorgan failure following ECMO decannulation. Given their deteriorating clinical trajectory and family-directed goals of care, they died after withdrawal of care. Respiratory support and clinical outcomes for each patient are summarized in Table 2.

### ECMO support

Three patients were placed on venovenous ECMO due to severe hypoxemic respiratory failure. The timing of cannulation and ECMO duration varied by patient. Anticoagulation strategies varied according to bleeding severity, with two patients restrictively anticoagulated using heparin or bivalirudin without hemorrhagic complications, while the third patient received no systemic anticoagulation due to ongoing hemoptysis. One patient's active clotting time (ACT) goal was 160–180 with heparin, and the other patient's aPTT goal was 60–70 with bivalirudin, with no reported hemorrhagic or thrombotic complications. All three patients were successfully decannulated from ECMO (Table 2).

### Therapeutic plasma exchange (TPE)

All patients had symptoms of hemoptysis upon admission/transfer, but DAH was not confirmed. Once DAH was confirmed (either by frank blood seen on BAL during bronchoscopy, active bleeding noted on catheterization, or diffuse opacification/haziness seen on imaging in the setting of active hemoptysis and respiratory distress), TPE was initiated within 24 h of confirmation for four patients. Two patients had a delay in TPE administration as it was thought that their symptomology was not severe enough, though both of those patients' hemoptysis persisted and worsened (one progressed to HFOV support), eventually requiring TPE initiation. One patient also received rituximab and had to wait 24 h before the first TPE. The total number of TPE sessions was 6.5 ± 2.6 (mean ± SD), with initiation between hospital days (HD) 2 and 12, depending on the patient. Replacement volume was usually 1.5 times total plasma volume (TPV) for the first treatment and followed by one-time TPV from the second. During ECMO, TPE is provided as a tandem therapy. Other patients had a hemodialysis catheter placement before TPEs. Fresh frozen plasma (FFP) was given as a replacement fluid for all the treatments, and citrate was used as local anticoagulation per protocol. The duration and spacing of sessions were determined based on clinical response and progression of respiratory symptoms by the primary ICU team, apheresis service, and rheumatologists. TPE was provided every other day for three patients and every day for the other three patients for the first three treatments. Where rituximab was administered, spacing was considered to minimize drug clearance by TPE, with at least 24 h between rituximab dosing and TPE initiation in all cases. No apparent complications were reported for TPE.

### Immunomodulatory treatment

All patients received high-dose glucocorticoids (HDGC) with a mean of 4.8 ± 2.2 doses (mean ± SD) as first-line therapy, along with cyclophosphamide. Additional immunomodulators used included rituximab (*n* = 5), mycophenolate mofetil (*n* = 3), hydroxychloroquine (*n* = 4), intravenous immunoglobulin (*n* = 3), and eculizumab (*n* = 1).

## Discussion

In this case series, we report six critically ill pediatric patients treated in our ICU for autoimmune DAH. Four patients had clinical concern or suspicion for SLE or AAV before admission, yet only two had received prior rheumatologic evaluation or immunosuppression. Morbidity was significant: five required mechanical ventilation, two required HFOV, and three required ECMO. All patients received pulse-dose corticosteroids and a median of five TPE sessions. Although TPE initiation generally occurred within 24 h of DAH confirmation, two patients experienced delays in TPE initiation because their symptoms were not severe, and rituximab was given. These two patients required higher respiratory support before the initiation of TPE. In all patients, alveolar hemorrhage and hypoxia improved after TPE and other immunomodulators administration. One mortality occurred due to refractory septic shock following ECMO decannulation.

### Autoimmune DAH

A major diagnostic challenge in pediatric autoimmune DAH lies in confirming the underlying autoimmune etiology, as hemoptysis can result from a broad differential. In our series, only two patients had a fully established autoimmune diagnosis before hospitalization. Delays in confirming DAH were commonly due to imaging initially interpreted as infectious or waiting for bronchoscopy to demonstrate ongoing bleeding. The prevalence of DAH—or severe DAH—as the initial manifestation of autoimmune disease is poorly documented (21, 22). Pediatric literature suggests that children may present more acutely and severely ill than adults (23), consistent with our cohort in which all six patients presented with or rapidly developed severe DAH. Adult data also highlight disease severity: in the PEXIVAS trial (24), 191 patients had DAH—164 (85.8%) were newly diagnosed with AAV, and 61 had severe DAH, with 29 (15%) requiring mechanical ventilation. Reported mortality for severe DAH in adult ICU populations ranges widely, with multicenter critical care cohorts demonstrating mortality rates exceeding 20% in mechanically ventilated patients (7). When limited specifically to autoimmune-associated DAH, adult studies report mortality of approximately 30%–40% in SLE-associated DAH and 15%–20% in AAV-associated DAH (11, 25, 26). Pediatric autoimmune-associated DAH mortality remains poorly defined. In comparison, our cohort demonstrated an in-hospital mortality of 16.6%, which is similar to adult autoimmune-associated DAH cohorts reporting mortality of approximately 15%–40%, while morbidity in our patients was substantial, with 83.3% requiring mechanical ventilation and 50% requiring ECMO. These findings reinforce that pediatric autoimmune DAH is a life-threatening condition that warrants aggressive management, including consideration of early TPE.

#### ECMO for autoimmune DAH

The use of ECMO in DAH remains challenging due to the bleeding risk associated with anticoagulation. According to the Extracorporeal Life Support Organization (ELSO), venovenous ECMO is recommended for severe, reversible respiratory failure refractory to medical management (27). In a retrospective study by Seeliger et al. (28), 28 patients had severe ARDS due to autoimmune DAH, with VV-ECMO implemented in 19 (68%). Mortality was high, with nine (47%) of those 19 patients suffering from hemorrhagic or septic complications. Registry-based analyses have further suggested that ECMO support in DAH secondary to autoimmune pulmonary vasculitides can achieve favorable survival, even with conservative anticoagulation strategies (29). In our cohort, three patients received ECMO with restrictive or no anticoagulation strategies. No ECMO-related hemorrhagic complications were observed in our cohort, supporting the feasibility of ECMO in selected pediatric DAH patients.

Data on pediatric patients with autoimmune DAH and ECMO support remains limited. One small case series reported eight patients under 19 years old receiving ECMO for pulmonary hemorrhage without mortality (6). Among them, six had systemic vasculitis SLE or AAV, and three received TPE. While all patients were anticoagulated, anticoagulation targets were lowered. Thus, our successful ECMO experience further supports that ECMO with restrictive anticoagulation is a viable option for refractory hypoxemia in pediatric DAH. Notably, TPE was performed safely even in patients requiring ECMO tandemly, without TPE-related hemorrhagic complications.

#### TPE in autoimmune DAH

TPE remains controversial in DAH, as current recommendations are based almost entirely on adult studies, many of which included patients with comparatively mild autoimmune disease (13, 15, 27, 28, 30, 31). The most recent ASFA guidelines assign TPE as a Category II, Grade 2C recommendation for SLE-associated DAH and Category III recommendation for AAV (Grade 1B for GPA/MPA; Grade 2C for EGPA) (14). As these guidelines are based almost entirely on adult studies (31), with comparatively mild pulmonary involvement, limiting their applicability to children with severe autoimmune DAH. Pediatric SLE is associated with higher mortality/more severe disease course than adult-onset SLE (32), and the pathophysiology of DAH in children may differ from adults (20, 33).

Recent evidence has also questioned the benefit of plasma exchange in autoimmune DAH. In a target trial emulation including 184 patients with severe ANCA-associated vasculitis-related DAH, the use of plasma exchange was not associated with improved short-term survival, with 30-day survival rates of 85% in patients receiving plasma exchange compared with 88% in those managed without it (24). However, the TPE group included patients who received TPE within 7 days, and some patients might have missed the critical timing for TPE. Also, albumin was used for replacement in 27% of patients. Our results suggest the necessity of further study, including the timing and replacement fluid of TPE for these patients. These findings also highlight the ongoing uncertainty regarding the benefit of TPE in autoimmune DAH and emphasize the need for disease-specific and population-specific evaluation, particularly in pediatric patients who often present with more severe pulmonary involvement than adult cohorts.

Currently, the standard treatment for autoimmune DAH involves high-dose glucocorticoids, followed by agents like cyclophosphamide or rituximab. TPE acts by removing pathogenic autoantibodies and immune complexes and provides clotting factors by using FFP as replacement fluid. Therefore, massive FFP transfusions could be accomplished without fluid overload, which can be beneficial for patients with active bleeding. In pediatric cases, vascular access can be a barrier to TPE initiation. In many cases, these children need a dialysis catheter placement, requiring a procedure under sedation, even though TPE can be performed in two large-bore peripheral IV accesses in adults (34). Treatment sequencing is another practical concern because TPE can remove circulating monoclonal antibodies like rituximab, potentially reducing their efficacy. In a pharmacokinetic study, the area under the curve (AUC) of rituximab exposure decreased by approximately 26% when TPE was performed 24 h after infusion and by 16% when performed at 72 h after infusion (30). To minimize this effect, we spaced TPE sessions at least 24 h after rituximab infusions in our patients to better preserve therapeutic exposure. One of our patients received rituximab soon after admission to the ICU and as such, it caused a delay in initiating TPE. We would strongly suggest considering TPE initiation first and then followed by rituximab if both are provided.

It should be noted that TPE does increase the risk of serious infection (relative risk of 1.27) based on a systematic review by Walsh et al. (35). Although serious infectious complications are a recognized concern following TPE, particularly in critically ill patients, vigilant surveillance allowed early detection and management in our cohort. TPE also theoretically introduces bleeding risks—with large-bore catheter placement in children (compared to possible placement with just two large-bore peripheral IVs in adults) and the removal of coagulation factors; these can be mitigated by using FFP for replacement in actively bleeding patients, as implemented in our cohort. Despite these concerns, multiple studies indicate that TPE is generally well tolerated, with serious complications being rare (12, 15, 36–40). Given the severity of pediatric DAH, the limitations of adult-derived guideline recommendations, and the favorable safety profile observed in our patients, early use of TPE in critically ill children with autoimmune DAH merits further evaluation.

### Limitations

Our study has limitations. As a retrospective review, care decisions were assessed based on documentation. We could not have identified some patients with autoimmune DAH due to missed diagnosis. This is a small study in a single center secondary to the uncommon nature of the condition. Our institutional practice may not reflect broader practice patterns. There could be a significant variation in practice over 11 years. All patients required ICU admission and received TPE along with heterogeneous regimens of immunomodulation therapies, therefore it is impossible to compare with treatment without TPE. The timing and number of TPE treatments are different among patients.

## Conclusion

Pediatric autoimmune DAH is frequently abrupt and severe, often presenting with hemoptysis and rapid respiratory failure, sometimes with inaugural autoimmune diseases. In children with confirmed or suspected autoimmune disease, aggressive diagnostic evaluation and early initiation of TPE—alongside immunosuppression and supportive therapies—may improve clinical outcomes. Given the immunomodulatory effects of TPE, careful monitoring for infectious complications is essential. Larger, prospective studies are needed to better define the role, timing, and safety of TPE in pediatric autoimmune DAH.

## Data Availability

The original contributions presented in the study are included in the article/Supplementary Material, further inquiries can be directed to the corresponding author.
